# Eltrombopag in Good's Syndrome

**DOI:** 10.1155/2014/172139

**Published:** 2014-10-19

**Authors:** Håvard Anton Kristiansen, Signe Spetalen, Yngvar Fløisand, Dag Heldal

**Affiliations:** ^1^Department of Internal Medicine, Innlandet Hospital Trust, 2629 Lillehammer, Norway; ^2^Department of Pathology, Oslo University Hospital, 0424 Oslo, Norway; ^3^Department of Hematology, Oslo University Hospital, Rikshospitalet, P.O. Box 4950, Nydalen, 0424 Oslo, Norway

## Abstract

Good's syndrome is a rare acquired immunodeficiency associated with thymoma. Eltrombopag is a thrombopoietin receptor agonist and has been shown to be a valuable supplement to the treatment of several types of refractory cytopenias. In this paper, we describe a male patient suffering from Good's syndrome with immune-mediated T-cell driven pancytopenia and absence of megakaryopoiesis. He was successfully treated with eltrombopag resulting in a multilineage clinical response.

## 1. Introduction

Good's syndrome is defined as an acquired immunodeficiency associated with thymoma. The association between the presence of thymoma and adult onset immunodeficiency was first recognized by Dr. Robert Good in 1954. The most consistent immunological abnormalities are hypogammaglobulinemia, reduced or absent B-cells, and inverted CD4/CD8 T-cell ratio [1, 2]. Eltrombopag, a thrombopoietin receptor agonist, is commonly used in the treatment of chronic immune thrombocytopenic purpura refractory to other treatment modalities [3]. Eltrombopag has also showed a multilineage effect improving hematopoiesis in refractory aplastic anemia with minimal toxicity [4]. We describe a patient suffering from Good's syndrome with immune mediated T-cell driven pancytopenia and absence of megakaryopoiesis, treated successfully with eltrombopag.

## 2. Case History

A 61-year-old male patient presented with symptoms of upper and lower respiratory tract infections with increasing frequency and severity over three years. Apart from hypogammaglobulinemia and a moderate neutropenia, a CT scan detected a thymoma, consistent with Good's syndrome. Thymectomy was performed, and he subsequently received immunoglobulin substitution. Three years after surgery, he developed transfusion dependent anemia and thrombocytopenia and was admitted to hospital with gastrointestinal hemorrhage and infections. There was no evidence of hypocellular MDS in the morphologic assessment of bone marrow aspirate. A bone marrow biopsy was hypercellular with T-cell lymphocytosis, absence of B-lymphocytes, normal erythropoiesis, megakaryopoiesis, and a left-shifted myelopoiesis with no evidence of aplastic anemia or myelodysplasia (MDS). A polyclonal T-cell receptor gamma gene rearrangement was found. The absolute number of T-cells in peripheral blood was not available. The CD4/CD8 ratio in the bone marrow was inverted. On suspicion of immune mediated pancytopenia, he received antithymocyte globulin from horse (3000 mg intravenously, days 1–4) followed by a 14-day course of prednisolone as prophylaxis against serum sickness and cyclosporine (5 mg/kg/day initially, then adjusted serum concentrations). At three months there was no clinical effect. A bone marrow biopsy revealed reduced cellularity from 80 to 10%, with hypoplastic myelo- and megakaryopoiesis, left-shifted erythropoiesis, and persistent T-cell lymphocytosis. Cyclosporine, s.c. immunoglobulin, and tranexamic acid were continued and G-CSF was introduced. Due to severe gingival hyperplasia and lack of effect on cytopenia, cyclosporine was discontinued after 7 months. The bone marrow remained severely hypocellular. As further therapeutic strategies, cyclophosphamide (100 mg daily orally) was given for 2 months and stopped due to increased transfusion dependency and lack of effect. Furthermore, high dose intravenous immunoglobulin was without impact. In addition, the patient received G-CSF 5 ug/kg subcutaneously three times weekly to alleviate neutropenia. PNH tests were negative, cytogenetics revealed a normal male karyotype, and there was no splenomegaly and no HLA-immunization.

**Figure 1:**
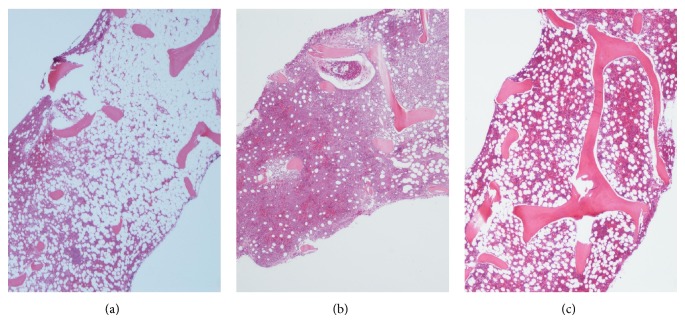
MGG stained bone marrow biopsies before (a), at 3 months (b) of, and at 12 months (c) of treatment (magnification ×10).

In a further therapeutic attempt, treatment with eltrombopag was initiated in a starting dose of 50 mg daily, escalating to 75 mg daily after two weeks. Before treatment, a bone marrow biopsy showed hyperplastic left-shifted myelopoiesis and aplastic megakaryo- and erythropoiesis (Figure 1(a)). One month after start of eltrombopag, he was no longer transfusion dependent.

**Figure 2:**
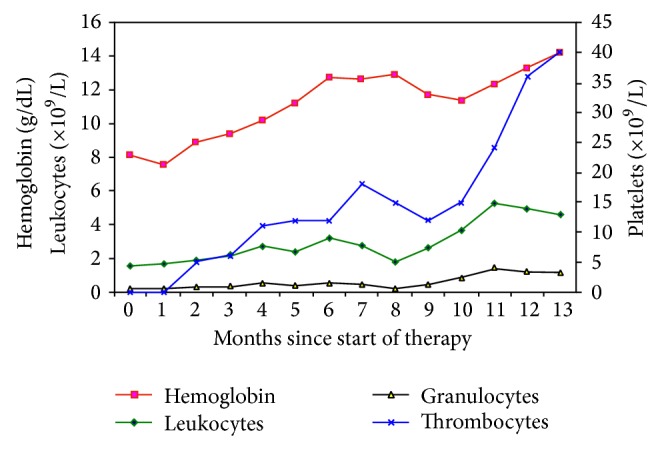
Development of hematological values before and during treatment.

At three-month evaluation the hemoglobin reached 11 g/dL and platelet count was 15–20 × 10^9^/L, compared to <8 g/dL and <10 × 10^9^/L, respectively, before treatment was started. Absolute neutrophil counts were normal and G-CSF was tapered to twice a week (Figure 2). A new bone marrow biopsy showed hyperplastic myelopoiesis, erythropoiesis, megakaryopoiesis with normal morphology, and fibrosis grade 1 (Figure 1(b)). His clinical condition remained stable and satisfactory.

At one year after initiation of eltrombopag 75 mg daily, the hemoglobin levels stabilized at 13 g/dL, the absolute neutrophil count is normalized, and the platelet count was >30 × 10^9^/L. Bone marrow biopsy showed hyperplastic erythropoiesis, slightly hyperplastic myelopoiesis, megakaryopoiesis with normal morphology, and fibrosis grade 0-1 (Figure 1(c)).

## 3. Conclusion

Eltrombopag has been shown to be a valuable supplement to the treatment armamentarium of refractory immune cytopenia. This is the first description of successful use of thrombopoietin receptor agonists in refractory cytopenia secondary to Good's syndrome, leading to a multilineage clinical response.
